# The Formation Mechanism of the Double Gas Layer in Gas-Assisted Extrusion and Its Influence on Plastic Micro-Tube Formation

**DOI:** 10.3390/polym12020355

**Published:** 2020-02-06

**Authors:** Tongke Liu, Xingyuan Huang, Cheng Luo, Duyang Wang

**Affiliations:** College of Mechanical and Electrical Engineering, Nanchang University, Nanchang 330031, China; 405928917131@email.ncu.edu.cn (T.L.); doyen_wang@126.com (D.W.)

**Keywords:** gas-assisted extrusion, double gas layer, micro-tube forming, shear thinning, viscous dissipation

## Abstract

The diameter of a micro-tube is very small and its wall thickness is very thin. Thus, when applying double-layer gas-assisted extrusion technology to process a micro-tube, it is necessary to find the suitable inlet gas pressure and a method for forming a stable double gas layer. In this study, a double-layer gas-assisted extrusion experiment is conducted and combined with a numerical simulation made by POLYFLOW to analyze the effect of inlet gas pressure on micro-tube extrusion molding and the rheological properties of the melt under different inlet gas pressures. A method of forming a stable double gas layer is proposed, and its formation mechanism is analyzed. The research shows that when the inlet gas pressure is large, the viscosity on the inner and outer wall surfaces of the melt is very low due to the effects of shear thinning, viscous dissipation, and the compression effect of the melt, so the melt does not easily adhere to the wall surface of the die, and a double gas layer can be formed. When the inlet gas pressure slowly decreases, the effects of shear thinning and viscous dissipation are weakened, but the gas and the melt were constantly displacing each other and reaching a new balanced state and the gas and melt changed rapidly and steadily in the process without sudden changes, so the melt still does not easily adhere to the wall of the die. Thus, in this experiment, we adjusted the inlet gas pressure to 5000 Pa first to ensure that the melt do not adhere to the wall surface and then slowly increased the inlet gas pressure to 10,000 Pa to reduce the viscosity of the melt. Lastly, we slowly decreased the inlet gas pressure to 1000 Pa to form a stable double gas layer. Using this method will not only facilitate the formation of a stable double gas layer, but can also accurately control the diameter of the micro-tube.

## 1. Introduction

Plastic micro-tubes are widely used in medicine, communication, automotive and other fields and they have high added value. In the past, the micro-tubes were extruded mainly using experimental methods and numerical simulation methods to analyze the melt flow behavior and extrusion swell in the micro-channel, and also, to analyze the influence of various factors on the melt flow stability and extrusion swell [1,2,3,4,5,6,7,8,9,10,11,12,13] in small-section flow channels. The numerical simulation of plastic micro-tube extrusion molding was carried out by Fu [14]. The influence of gas injection pressure on micro-tube cross-section size, pressure field, and velocity field were analyzed, and a reasonable gas injection pressure range was determined. Some scholars [15,16,17,18,19,20,21,22,23] also analyzed micro-tube extrusion dies while researching micro-tube extrusion, but they have not solved the problem of extrusion swelling [24,25,26,27,28,29,30,31,32,33,34,35], melt fractures, and the distortion of micro-tube extrusion. The application of gas-assisted extrusion technology [36,37] could eliminate these problems to some extent. Gas-assisted extrusion molding was first proposed by Liang R.F. [38]. Gas was used as an assisted process in the polymer extrusion molding process by means of a slit inlet. Xu [39] used finite element simulation software to simulate the extrusion swell of extruded products with different extrusion flows, tube diameters, wall thicknesses, and gas-assisted lengths for the round-tube gas-assisted extrusion process. As the technology has matured, the slip phenomenon has been confirmed by many scholars [40,41,42,43,44,45,46,47,48]. However, the gas-layer of the numerical simulation of these gas-assisted extrusion was simplified as a completely slip-free surface boundary condition of the wall surface without friction, and only the effect of gas on reducing the frictional resistance between the die and melt was considered, but the effect of gas compressibility on extrusion was ignored. Until Ren [49,50,51,52,53,54] established a gas/melt two-phase flow model and designed a double-layer gas-assisted extrusion die, gas was considered to be one phase while evaluating the compressibility of the gas, and the effect of the gas layer inside the die on micro-tube formation was also investigated. The prototype of double-layer gas-assisted extrusion was thus established. However, in past experiments and numerical simulations of double-layer gas-assisted extrusion, the size of the extruded tubes was large, and the melt was not easily broken, so the inlet gas pressure was relatively simple, the stable double gas layer was relatively easy to form. In the past, only the compressibility of the gas was considered, however, the compressibility of the melt was ignored. Regarding the extrusion of micro-tubes with very thin wall thicknesses, the wall is easily broken and it adhered to the wall surface of the die, and the stable double gas layer was relatively difficult to form, so a method for forming a stable double gas layer and suitable inlet gas pressure must be found. In this experiment and simulation, the compressibility of both the gas and the melt were considered to accurately analyze the influence of inlet gas pressure on melt forming. The formation mechanism of a stable double gas layer and its influence on plastic micro-tube formation have also been presented.

## 2. Materials and Methods

### 2.1. Materials

The polymer is polypropylene (PP, K9015), which was purchased from Sinopec Yangzi Petrochemical Co., Ltd (Nanjing, China). The auxiliary gas is dehumidified and compressed air. The physical parameters of the polypropylene are shown in Table 1.

### 2.2. Extrusion Experiments

**Figure 1:**
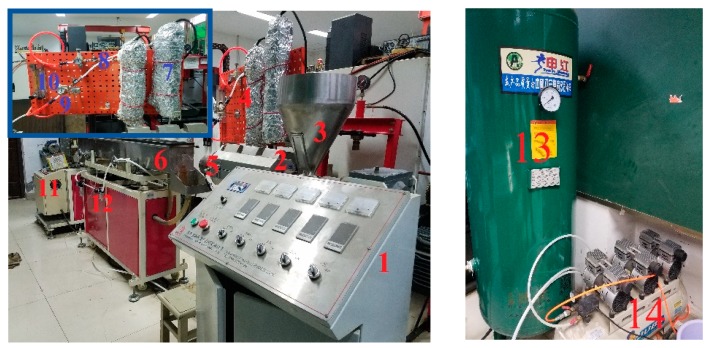
Experimental machine. **1**—Extruder console; **2**—Single-screw extruder; **3**—Extruder feed shake; **4**—Gas unit console; **5**—Die; 6—Cooling sink; **7**—Gas heating device; **8**—Gas pipeline; **9**—Pressure controller; **10**—Rotameter; **11**—pulling system; **12**—pressure regulating filter; **13**—Gas storage tank; **14**—Air compressor.

The experimental machine is shown in Figure 1. The single-screw extruder used in the experiment is a SJ-25 machine with a screw diameter of 25 mm (the length to diameter ratio is 25:1, with a power of 2.2 kW) which was purchased from Dongguan Huaxi Plastic Machinery Co., Ltd. (Dongguan, China). The pulling system (GRQ-25PVC) was designed by Dongguan Huaxi Plastic Machinery Co., Ltd. (Dongguan, China). The single-screw air-cooled series air compressor (MODEL 0G06F) was produced by Shanghai Jialishi Machinery Co., Ltd. (Shanghai, China). The high-pressure gas storage tank (model 1V −3/8, volume 1.0 m^3^) was designed by Dingxin pressure vessel Co., Ltd. (Linyi, China). The rotameters (LZB-40) were purchased from Yuyao silver ring flow Instrument Co., Ltd. (Yuyao, China). The pressure controllers (Y-40) was produced by Shanghai Tianchuan Instrument Factory (Shanghai, China). The gas heating device was designed by Shanghai laiheng Electric Appliance Co., Ltd. (Shanghai, China). The pressure regulating filter (AFC2000) was produced by AirTac Co., Ltd. (Taipei, China). Gas pipelines were used in this experiment, where the air compressor can discharge up to 0.8 m^3^/min, the maximum pressure is 0.7 MPa. The gas heating device is composed of a thermocouple and temperature controller (XMT-101, Jia Ming Instrument Co., Ltd., Shanghai, China) to achieve gas temperature control.

**Figure 2:**
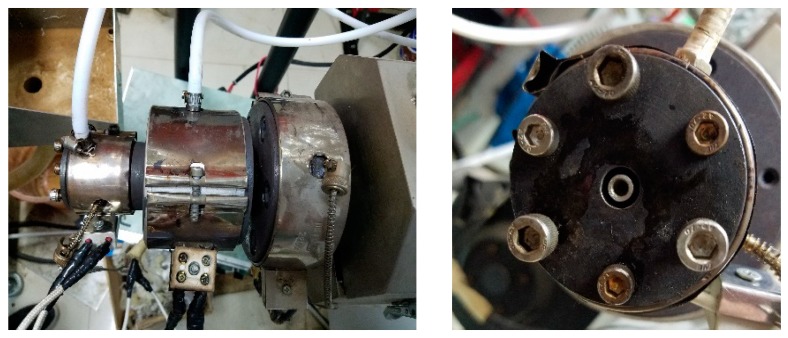
Double-layer gas-assisted extrusion die. The left one is physical assembly picture of the die, the right one is physical picture at the die exit.

**Figure 3:**
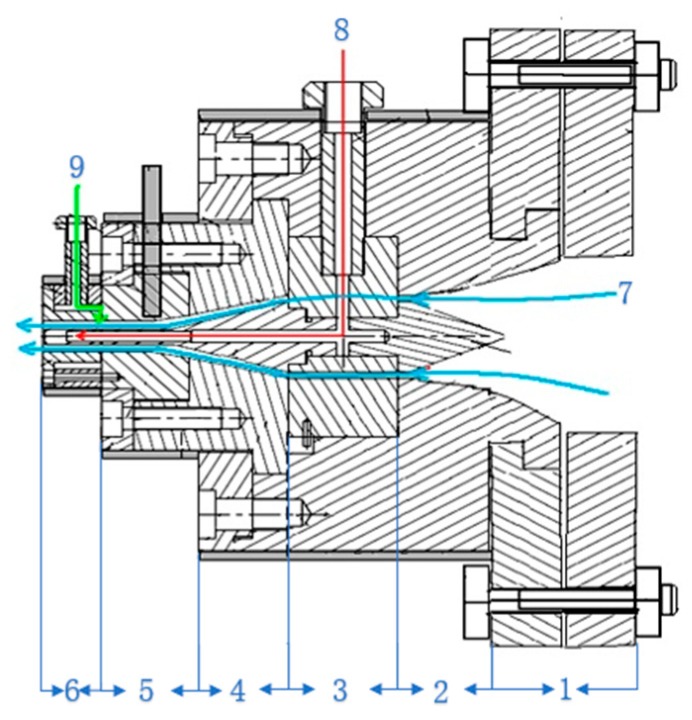
Structural diagram of the die. **1**—Melt inlet section; **2**—Melt split section; **3**—Melt advective section; **4**—Melt compressive section; **5**—No gas-assisted forming section; **6**—Gas-assisted forming section; **7**—Melt flow direction; **8**—Inner gas layer flow direction; and, **9**—Outer gas layer flow direction.

The double-layer gas-assisted extrusion die is the most important part of the experimental equipment, it was designed and manufactured by our research team and it is shown in Figure 2. The structure of the die is shown in Figure 3. It can be seen that the inner gas layer enters the die from the gas channel of the ventilation bolt, passes through the gas channel of the split cone bracket connecting component, enters the gas channel in the middle of the mandrel, and then flows out from the gas groove on the side of the mandrel. In this way, the inner gas-assisted layer between the outer wall of the mandrel and the inner wall of the melt is formed. The outer gas layer enters the gas chamber from the gas channel of the ventilation bolt and then enters the channel of the die from the gas intake gap, thereby forming the outer gas-assisted layer between the outer wall of the melt and the inner wall of the die channel. The melt flows through the inlet section, split section, advective section, compressive section, no gas-assisted forming section, and gas-assisted forming section in sequence, then, the formed micro-tube can be obtained after cooling and sizing. The experiment used a horizontal extruded method to freely extrude the melt.

The processing parameters are shown in Table 2. First, we tested the influence of the opening order between the inlet gas switch and the extruder on the formation of the double gas layer. Then, we tested the effects on the formation of the double gas layer under different inlet gas pressures (100 Pa and 15,000 Pa were set in the experiment) and analyzed the effect on micro-tube formation. At last, we tested the effect on the formation of the double gas layer when the inlet gas pressure was adjusted from 5000 to 10,000 Pa and then adjusted to 1000 Pa. Next, we analyzed how these variations affected the formation of micro-tubes.

### 2.3. Numerical Simulation Theories and Methods

#### 2.3.1. Geometric Models and Finite Element Models

**Figure 4:**
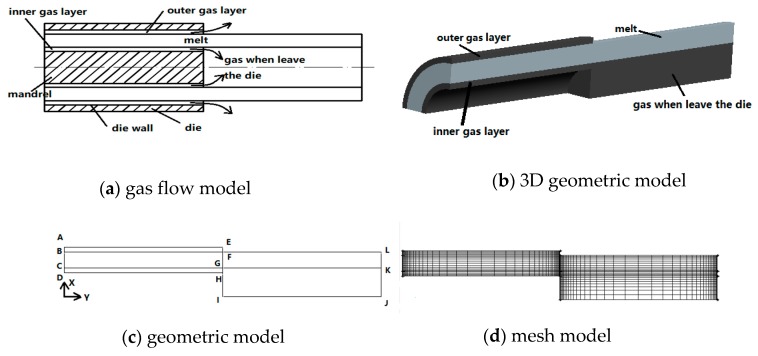
Geometric and mesh models. (**a**) gas flow model, (**b**) 3D geometric model, (**c**) geometric model, (**d**) mesh model.

It can be seen from Figure 4a that. in the die, there is an inner gas layer and an outer gas layer, respectively, flowing close to the inner and outer walls of the micro-tube, and the inner gas layer flows in the micro-tube cavity after leaving the die. The outer gas layer blows into the air after leaving the die. A 3D geometric diagram is shown in Figure 4b. Since the plastic micro-tubes are axisymmetric, in order to save computational memory consumption and improve finite element calculation efficiency, a two-dimensional model diagram along the flow channel direction is used to calculate, as shown in Figure 4c, using a 2D 1/2 axisymmetric method to calculate. The entire die flow path width AD is 1.2 mm, wherein the melt width BC is 1 mm, the outer gas layer width AB is 0.1 mm, the inner gas layer width CD is 0.1 mm, and the flow radius KJ in the microcavity after the inner gas layer leaves the die is 1.5 mm. In the figure, BLKC is the melt region (BFGC is the melt region inside the die, FLKG is the melt region downstream the die exit), AEFB is the outer gas layer, CGHD is the inner gas layer, and GKJI is the gas flow area in the micro-tube after the inner gas layer leaves the die. The length AE of the gas layer inside the die is 10 mm and the length FL of the melt downstream the die exit is 10 mm. Assuming that the melt and gas flow directions are in the Y-axis direction of the coordinate, the finite element mesh diagram is shown in Figure 4d. In order to improve the calculation accuracy, we implemented grid optimization at the entrance, exit, and end of the die, and the total number of grids was 1764.

#### 2.3.2. Control Equations and Constitutive Equations

According to the flow characteristics of the polymer melt and gas in the die during gas-assisted extrusion, the following assumptions are made:

(1) Under micro-scale conditions, the polymer melt is regarded as a compressible non-Newtonian viscoelastic fluid.
(2) Under micro-scale conditions, the gas is regarded as a compressible Newtonian fluid.
(3) The melt and the gas are considered to be a steady laminar flow.
(4) The effect of the inertial force and gravity on the flow of the two fluids are ignored.
(5) The relative slip of the gas with the die wall and the melt are ignored.
(6) The osmosis of gas molecules into the melt is ignored.

The governing equations for the melt and gas are as follows:

Continuity Equation:

(1) $$\nabla \cdot (\rho_{k}v_{k})=0$$

Momentum Equation:

(2) $$\rho_{k}v_{k}\cdot \nabla v_{k}+\nabla p_{k}-\nabla \tau_{k}=0$$

Energy Equation:

(3) $$\rho_{k}C_{pk}v_{k}\cdot \nabla T_{k}-k_{k}\nabla^{2}T_{k}=\tau_{k}:\nabla v_{k}$$

where ∇ is a Hamiltonian, $\rho_{k}$ is the density, $v_{k}$ is the velocity vector, $p_{k}$ is the pressure, $\tau_{k}$ is the deviatoric stress tensor, $C_{pk}$ is the specific heat at constant pressure, and $T_{k}$ is the temperature, where k=Ι,Π represents the melt and the gas, respectively.

The Cross-WLF simple viscoelastic constitutive equation is used to describe the flow characteristics of the polymer melt. The Cross-WLF constitutive equation is as follows:

(4) $$\eta \left(\overset{•}{\gamma },T_{k},P_{k}\right)=\frac{\eta_{0}\left(T_{k},P_{k}\right)}{1+{\left(\frac{\eta_{0}\overset{•}{\gamma }}{\tau *}\right)}^{1-n}}$$

(5) $$\eta_{0}\left(T_{k},P_{k}\right)=D_{1}\exp\left[\frac{A_{1}\left(T_{k}-T*\right)}{\tilde{A_{2}}+\left(T_{k}-T*\right)}\right]$$

(6) $$T*=D_{2}+D_{3}P_{k}$$

(7) $${\tilde{A}}_{2}=A_{2}+D_{3}P_{k}$$

where $\tau^{*}$ is the material constant, n is the non-Newtonian index, and $\eta_{0}$ is the zero shear rate viscosity; $\overset{•}{\gamma }$ is the shear rate; T∗ is the glass transition temperature of the material; ${\tilde{A}}_{2}$ is the model correction constant; $D_{1}$, $A_{1}$ and $A_{2}$ are the model constants; and, $D_{2}$ is the glass transition temperature at low pressure. $D_{3}$ is the pressure influence coefficient, which indicates the pressure dependence of viscosity.

For the gas, the constitutive equation is

(8) τ=2ηD

The gas deformation velocity tensor equation is

(9) $$D=\frac{1}{2}(\nabla v+\nabla^{T}v)-\frac{1}{3}\nabla v\delta$$

where δ is a second order unit tensor.

Due to the micro-scale conditions, the melt density under non-isothermal conditions is greatly affected by pressure and temperature. The relationship of the melt density with pressure and temperature can be expressed by the following equation:

(10) $$\rho =\frac{1}{V(T,P)}$$

where V(T,P) is the specific volume of melt at pressure P and temperature T; The V(T,P) can be expressed by the Tait equation [55,56]:

(11) $$V(T,P)=V0(T)\left(1-C\ln\left(1+\frac{P}{B(T)}\right)\right)+V1(T,P)$$

where V0 is the specific volume of the melt at zero pressure; C is a universal constant, and the value for the polymer is 0.0894; and, B is a parameter of the reacting material’s pressure sensitivity.

When the polymer is in a molten state (T>b5+b6P),

(12) V0=b1+b2(T−b5)

(13) B(T)=b3exp(−b4(T−b5))

(14) V1=0

where b1 is the specific volume by extrapolating the zero pressure isobaric curve to the transition temperature; b3 and b4 are the characteristics of B(T) in solid and molten states; b5 is the transformation temperature of the specific volume measured at zero pressure; and, b6 is the change in the linear temperature of the transition temperature due to pressure.

Since the gas is a compressible fluid, in order to make a solution of the partial differential equations of the gas, the density-pressure method is used in this paper—that is, a pressure variable is used to replace the density variable in the governing equation according to the relationship between the gas density and the pressure. This paper introduces the gas state equation:

(15) p=ρRT

where R is a gas constant and its value is R=287J/kg⋅K.

#### 2.3.3. Boundary Conditions

A two-dimensional model of plastic micro-tube dual-gas-assisted extrusion along the flow path based on the gas-liquid-gas mode is shown in Figure 4c, according to this model, the following boundary conditions are set:

(1) Inlet boundary: BC is the melt inlet boundary, AB and CD are gas inlet boundaries. According to the experiment [57,58,59,60], the gas temperature should be equal to or slightly higher than the melt temperature, so the temperature at the gas inlet boundary is still set to 190 °C.
(2) Wall boundary: AE is the wall surface of the die, DH and HI are the wall surfaces of the core rod. The die wall temperature is set to 190 °C, which is the same as the melt temperature.
(3) Symmetry boundary: The Y-axis (IJ in the figure) is the symmetry boundary of the model.
(4) Interface: BF and CK are the boundaries between the gas and the melt. Due to the continuity of the interface temperature, the temperature conditions are the same as the boundary of the interface.
(5) Free surface boundary: EL is the free surface boundary of the melt after the extrusion die. Since the free surface boundary is in direct contact with the outside in order to generate a heat convection exchange, the temperature boundary is set to a heat flux condition, and the influence of the melt heat radiation is ignored.
(6) End boundary: LK is the melt end boundary; KJ and EF are the exit boundaries of the inner and outer gas layers, respectively. Since the melt and gas outlet temperatures are unknown, both fluid temperatures are set to the temperature outflow condition.

#### 2.3.4. Setting of the Numerical Simulation Parameters

The numerical simulation parameters [56] of the melt and gas are shown in Table 3, where the physical property parameters of the air are obtained by referencing data given at a standard atmospheric pressure and at a temperature of 190 °C. The parameters for Tait equation [56] used in the numerical simulation are shown in Table 4.

#### 2.3.5. Software and Numerical Methods

In this numerical simulation, GAMBIT was used to build geometric models and finite element models, the ANSYS PLOYFLOW module was used for finite element simulation, and the ANASYS CFD-POST module was used for post-processing analysis. 

In order to facilitate the solution of the equation, a 2D 1/2 axisymmetric geometry model was used to perform the finite element calculation, while the Elastic Viscous Split Stress method and Streamline Up-winding method were combined to solve the equation. In order to make the finite element calculation finally converge, the melt relaxation time and the motion boundaries were set by the evolution method, and an iterative calculation was performed by the Galerkin method. For the free surface boundary, remeshing was performed using the Spines method.

In order to explain the formation mechanism of the stable double gas layer and the influence of the gas layer on the formation of the melt extrusion, in the experiment, four different inlet gas pressures (100, 1000, 5000, 10,000 Pa) were set. The variables of pressure, temperature, density, shear rate, and viscosity of the melt were obtained and analyzed.

## 3. Results and Discussion

Because the formation of a stable double gas layer is closely related to the viscosity of the melt inside the die, in the simulation, we analyzed the factors that affect the viscosity change, such as the pressure of the melt, the temperature of the melt, and the shear rate. In the experiments, we analyzed the formation method and formation mechanism of the stable double gas layer in combination with the change of the shear rate and the melt viscosity.

### 3.1. Simulation Results and Analysis

#### 3.1.1. The Results and Analysis of Pressure

**Figure 5:**
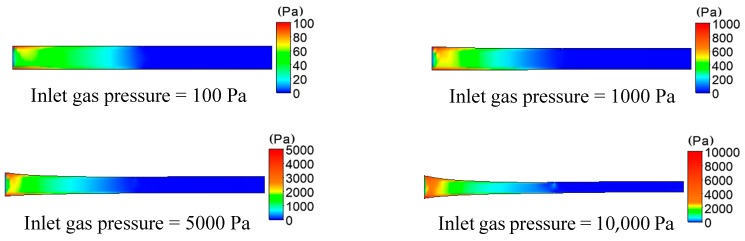
Distributions of pressure under different inlet gas pressures.

The distributions of pressure in the formation of plastic micro-tubes using gas-assisted extrusion are shown in Figure 5. 

It can be seen from Figure 5 that at the inlet of the die, the gas of the gas-assisted layer begins to act on the melt, and the pressure of the melt rapidly increases. Moreover, the closer to the surface of the melt, the greater the pressure. Then, a large pressure drop is generated near the inlet of the die. As the inlet gas pressure increases from 100 to 10,000 Pa, the pressure that is generated on both sides of the wall of the plastic micro-tube gradually increases from 100 to 10,000 Pa, and the pressure drop also gradually increases from 30 to 7000 Pa. When the inlet gas pressure increases to 10,000 Pa, the pressure distribution gradient can still be seen beyond the exit die. There is a small but significant pressure drop in the melt at the exit of the die, and the greater the gas inlet pressure, the more obvious the effect of the outer gas layer on the outer wall of the micro-tube.

#### 3.1.2. The Results and Analysis of Temperature

**Figure 6:**
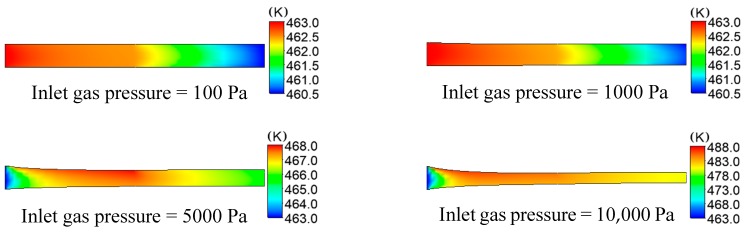
Distributions of temperature under different inlet gas pressures.

**Figure 7:**
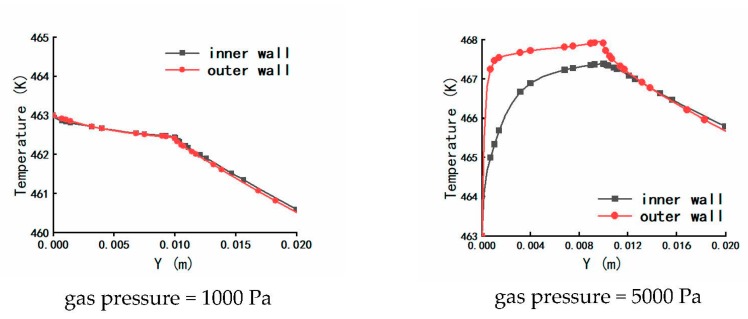
Distributions of temperature on the inner and outer wall of the melt.

**Figure 8:**
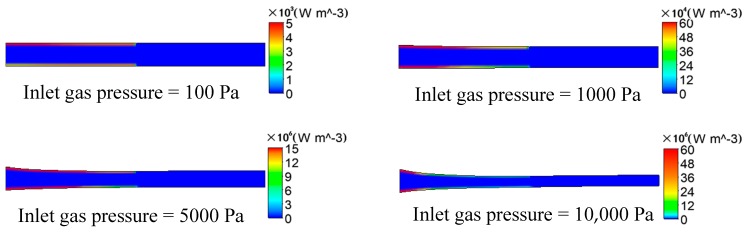
Distributions of viscous heating under different inlet gas pressures.

The distributions of temperature on plastic micro-tube formation while using gas-assisted extrusion are shown in Figure 6. Distribution has an important influence on the formation of the stable double gas layer when the inlet gas pressure is 1000 and 5000 Pa, so we focused on the analysis of the two inlet gas pressures. The distributions of temperature on the inner and outer wall of the melt along the axial direction are shown in Figure 7. The distributions of viscous heating on the formation of plastic micro-tube using gas-assisted extrusion are shown in Figure 8. 

It can be seen from Figure 6 and Figure 7 that when the inlet gas pressure is 100 or 1000 Pa, the temperature of the melt inside the die is maintained at approximately 463 K, the temperature of the melt downstream the die exit almost linearly declines from 462.5 to 460.5 K, and the temperature drop rate of the outer wall of the melt is slightly larger than that of the inner wall. When the inlet gas pressure is 5000 or 10,000 Pa, the temperature of the melt inside the die increases from 463 to 468 K or 463 to 488 K, respectively; and the temperature of the melt increases more rapidly the closer it is to the outer wall. The temperature of the melt downstream the die exit continuously decreases, and the temperature declines at a faster rate at the outer wall of the melt than at the inner wall.

It can be seen from Figure 8 that, when the inlet gas pressure is 100 or 1000 Pa, the viscous dissi Pation effect of the melt is not obvious, and the maximum generated viscous heating is 5 × 10^3^ or 6 × 10^5^ W/m^3^, respectively. However, as the inlet gas pressure increases from 100 to 10,000 Pa, the viscous dissipation effect of the melt is constantly enhanced, and the maximum generated viscous heating is 15 × 10^6^ or 6 × 10^7^ W/m^3^, respectively. The generated viscous heating and the generated heating of compressed melt cause the temperature of the surface of the melt to rise continuously, increasing by nearly 25 K when the inlet gas pressure is increased to 10,000 Pa. The closer to the surface of the melt, the more obvious the viscous heating generated. Gas still flows in the microcavity of the micro-tube downstream the die exit, so viscous heating still exists in the microcavity and it affects the inner wall surface of the melt, which makes the rate at which the temperature decreases at the outer wall of the melt greater than that of the inner wall.

#### 3.1.3. The Results and Analysis of Shear Rate

**Figure 9:**
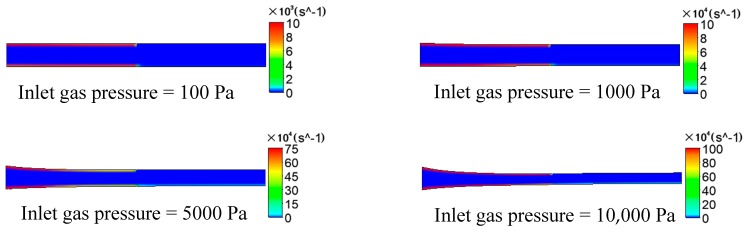
Distributions of the shear rate under different inlet gas pressures.

**Figure 10:**
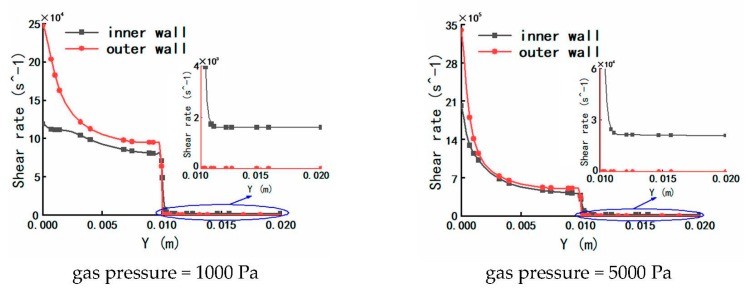
Distributions of shear rate on the inner and outer wall of the melt.

The distributions of the shear rate on plastic micro-tube formation while using gas-assisted extrusion are shown in Figure 9. For the analysis under 1000 Pa and 5000 Pa, the distributions of the shear rate on the inner and outer walls of the melt along the axial direction are shown in Figure 10, and the distributions of the shear rate downstream the die exit are magnified.

It can be seen from Figure 9 that at the interface between the gas and the melt, whether inside the die or downstream the die exit, the gas has a significant shear effect on the surface of the melt, and the closer to the surface of the melt, the larger the value of the shear rate. Inside the melt, however, there is almost no shear effect. As the inlet gas pressure increases from 100 to 10,000 Pa, the magnitude of the shear rate increases by more than 100 times.

It can be seen from Figure 10 that along the Y axis, the shear rate on the outer and inner wall of the melt gradually decreases and remains approximately unchanged near the exit of the die, which reflects the process of the gas and the melt constantly displacing and reaching balance. When the inlet gas pressure is 1000 Pa, the maximum shear rate on the inner wall and the outer wall of the melt is 1.2 × 10^5^ and 2.5 × 10^5^ s^−1^, respectively; when the inlet gas pressure is 5000 Pa, the maximum shear rate on the inner wall and the outer wall of the melt is 2 × 10^6^ and 3.4 × 10^6^ s^−1^, respectively. The outer wall surface has a much larger shear rate than the inner wall surface at the same Y coordinate, which indicates that the outer gas layer has a more significant shearing effect on the melt than the inner gas layer under the same inlet gas pressure. There is still significant shear effect at the inner wall surface of the melt downstream the die exit, while the shear rate on the outer wall is zero, with almost no shearing effect.

#### 3.1.4. The Results and Analysis of Viscosity

**Figure 11:**
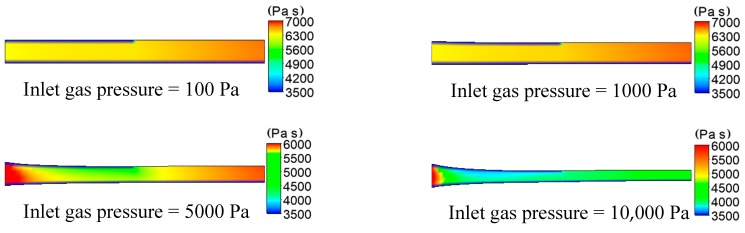
Distributions of viscosity under different inlet gas pressures.

Due to the changes of the pressure, the temperature and the shear rate as described above, the changes of viscosity on plastic micro-tube formation using gas-assisted extrusion are shown in Figure 11.

It can be seen from Figure 11 that when the inlet gas pressure is 100 or 1000 Pa, the viscosity inside the melt is almost constant at 6300 Pa·s inside the die, and the viscosity inside the melt increases from 6300 to 7000 Pa·s downstream the die exit. When the inlet gas pressure is 5000 Pa or 10,000 Pa, the viscosity inside the melt decreases, respectively, from 6000 s to 5000 Pa·s or 6000 to 4000 Pa·s inside the die. The closer the melt is to the outer wall, the faster the viscosity decreases; the viscosity of the melt increases, respectively, from 5000 P to 5800 Pa·s or 4000 to 4800 Pa·s downstream the die exit. The viscosity of the melt at the inner and outer wall surfaces is low due to the effects of shear thinning and viscous dissipation.

### 3.2. Experimental Results and Analysis

In the experiment of double-layer gas-assisted extrusion micro-tubes, it was found that after first opening the extruder, due to the high viscosity of the melt, the melt adhered to the wall inside the die, and blocked the gas-assisted flow channels. When the inlet gas switch turned on, the gas could not blow the melt completely from the wall surface of the die, so it was difficult to form a stable gas layer. Thus, this gas-assisted method was not feasible.

**Figure 12:**
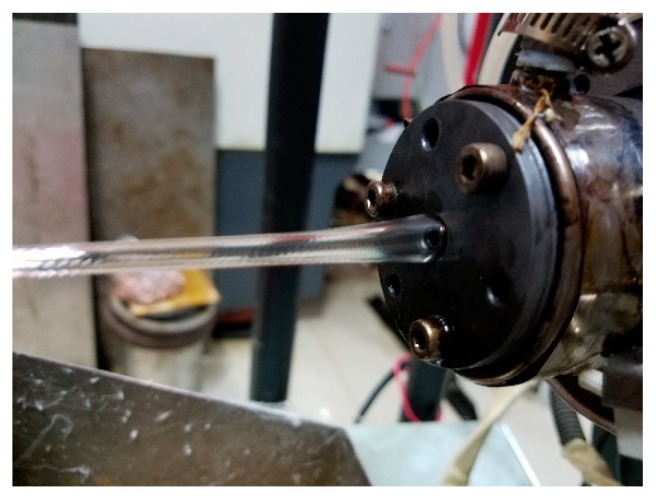
Micro-tube formation under 100 Pa.

We turned on the inlet gas switch and adjusted the inlet gas pressure to 100 Pa first, so a stable double gas layer could be formed. At this stage, the screw speed of the extruder was 0. Then, we adjusted the screw speed to increase slowly. Because the gas inlet pressure was much lower than the pressure when the melt entered the gas-assisted section of the die, even if the screw speed was 1 r/min, the melt would completely extrude the gas out of the die, and block the gas-assisted flow channels inside the die, which made the double-layer gas-assisted fail. Once the melt came into contact with the inner wall surface inside the die, it could adhere to the inner wall surface. Due to the viscosity of the melt adhered on the inner wall of the die was very high, then, even if the inlet gas pressure was increased continuously, the melt would not be easily displaced to form a stable double gas layer. Moreover, the wall of the micro-tube had continuous rugged corrugations. The resultant effect on the formation of the micro-tubes is shown in Figure 12 when the inlet gas pressure is 100 Pa.

**Figure 13:**
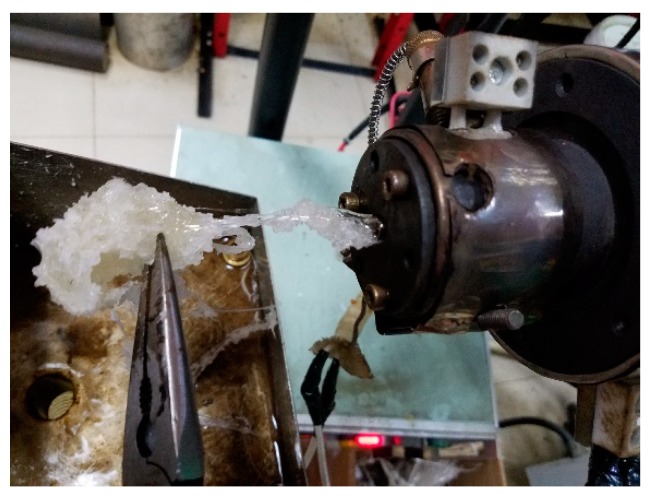
Micro-tube formation under 15,000 Pa.

We first turned on the gas inlet switch and adjusted the gas inlet pressure to 15,000 Pa. Although a stable double gas layer was not easily formed at this stage, the gas-assisted flow channels were not blocked when the screw speed was 5.5 r/min. However, in the gas-assisted extrusion, it was found that the melt was completely displaced from the inner wall surface by the gas inside the die, and the formed micro-tubes could not be obtained. Moreover, the broken melt would adhere to the inner wall inside the die. Then, the gas could not blow the broken melt completely from the wall surface of the die even if the inlet gas pressure was slowly reduced, it was very difficult to achieve a stable double gas layer. The resultant effect on the formation of the micro-tubes is shown in Figure 13 when the inlet gas pressure is 15,000 Pa.

**Figure 14:**
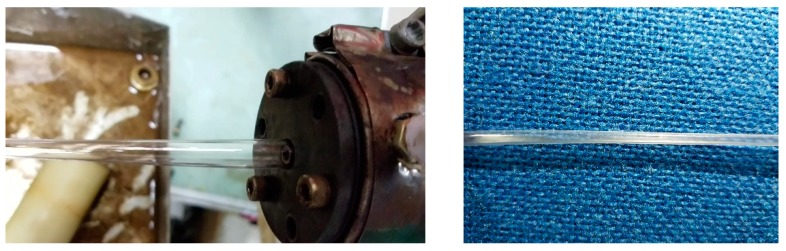
Micro-tube formation under 5000 Pa.

**Figure 15:**
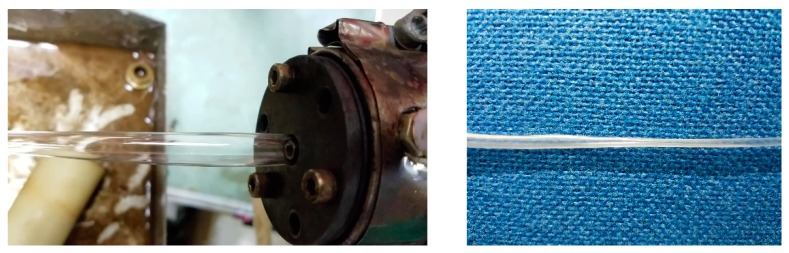
Micro-tube formation under 10,000 Pa.

We first turned on the inlet gas switch and adjusted the gas inlet pressure to 5000 Pa. At this stage, an unstable double gas layer was formed. Then, we adjusted the screw speed to increase slowly to 5.5 r/min. The gas was squeezed, but the melt did not completely squeeze the gas out of the die. The resultant effect on the formations of the micro-tubes is shown in Figure 14 when the inlet gas pressure is 5000 Pa. According to the results of the above viscosity analysis, the viscosity inside the melt was still large at this stage. In order to prevent the melt from completely extruding the gas out of the gas-assisted section of the die again, we slowly increased the gas inlet pressure and took care not to increase the pressure so fast that is displaced the melt, otherwise the cracked melt would adhere again to the inner wall surface of the die. After reaching a certain inlet pressure (up to 10,000 Pa in the experiment), the viscosity at the interface between the gas and the melt dropped below 3500, and the viscosity inside the melt dropped below 4000. Although the wall thickness of the micro-tubes became very thin, the wall surface of the micro-tubes had continuous rugged corrugations, and the diameter of the micro-tubes was constantly changing, and the melt would not adhere the wall inside the die at all. The resultant effect on the formation of micro-tubes when the inlet gas pressure is 10,000 Pa is shown in Figure 15.

**Figure 16:**
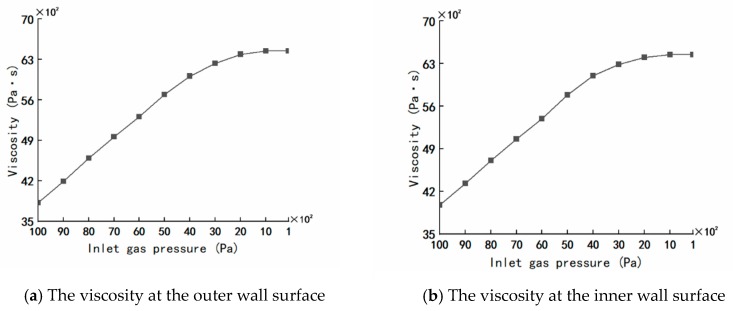
The viscosity at the wall surface of the melt under different inlet gas pressures. (**a**) The viscosity at the outer wall surface; (**b**) The viscosity at the inner wall surface.

After an observation period, we adjusted the gas inlet pressure to slowly decrease. According to the analysis of the shear rate as above, at the exit of the die, the melt and the gas have been displaced completely and then reached a steady state, so we analyzed the distributions of the viscosity at the wall surface of the melt under different inlet gas pressures, and the results of the analysis are shown in Figure 16. It can be seen that the trend of the viscosity changes is approximately the same whether it is at the inner wall surface or outer wall surface of the melt. We adjusted the inlet gas pressure to gradually decrease from 10,000 to 4000 Pa, the viscosity increased linearly from below 3900 to above 6000 Pa. Although the inlet gas pressure decreased and the viscosity increased, the gas and the melt were constantly displacing each other and reaching a new balanced state. During this process the gas and melt changed rapidly and steadily without sudden changes, and we found that the wall thickness of the micro-tubes gradually increased, the corrugations on the wall of the micro-tubes gradually disappeared, and the diameter of the micro-tubes tended to be stable.

**Figure 17:**
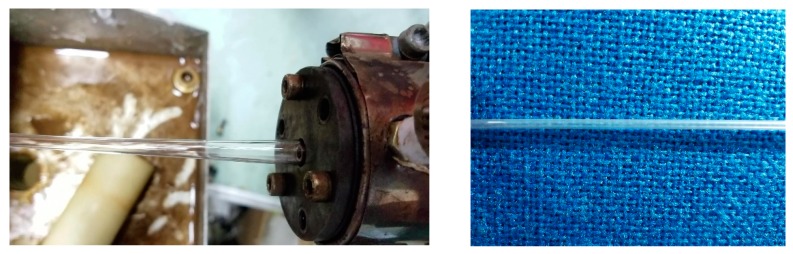
Micro-tube formation under 1000 Pa.

According to the analysis of the shear rate, as above, when the inlet gas pressure is 1000 Pa, the value of the shear rate can meet the processing requirements of stable gas-assisted extrusion, but it cannot meet the processing requirements when the inlet gas pressure exceeds 1000 Pa. Furthermore, the larger the inlet gas pressure, the larger the shear rate and the more significant the shearing effect of the gas on the melt. So then we slowly adjusted the gas inlet pressure to 1000 Pa, although the inlet gas pressure decreased, the viscosity did not change much, especially when the inlet gas pressure was less than 3000 Pa. Regardless of the value of the inlet gas pressure, the change of the viscosity was 100 Pa·s at the interface of the gas and the melt, the formed balance state between the melt and the gas was more stable and the formation of micro-tubes was optimized. The resultant effect on the formation of micro-tubes is shown in Figure 17. It can be seen that when the inlet gas pressure is 1000 Pa, the obtained micro-tube not only has a suitable diameter but also a smooth surface without ripples.

If the adjusted inlet gas pressure continued to decrease, because the gas inlet pressure was much lower than the pressure when the melt entered the gas-assisted section of the die, the melt would completely extrude the gas out of the die and the gas flow channels inside the die would be blocked. It has been experimentally verified that a stable double gas layer can be formed by such a method and a suitable diameter and surface quality of the micro-tubes can be ensured.

## 4. Conclusions

In this study, we used double gas layer gas-assisted technology for the extrusion of micro-tubes. Specifically, we adjusted the inlet gas pressure to 5000 Pa to ensure that the melt not only did not adhere to the wall’s surface, but it also did not break, and an unstable double gas layer was formed. Then, we slowly adjusted the inlet gas pressure up to 10,000 Pa to enhance the effects of shear thinning and viscous dissipation, and the compression effect of the melt. The shear rate and the viscous heating on the surface of the melt increased to more than 10^6^ s^−1^ and 6 × 10^7^ W/m^3^, respectively; the temperature increased by nearly 25 K, so the viscosity at the inner and outer wall surfaces of the melt decreased to less than 4000 Pa·s, and the double gas layer became more unstable. Lastly, we slowly adjusted the inlet gas pressure to 1000 Pa to ensure that the shear rate decreased to less than 10^5^ s^−1^ to meet the processing requirements. Even if the inlet gas pressure decreased, the effects of shear thinning and viscous dissipation were weakened and the viscosity at the interface of the gas and the melt increased, and the gas and the melt were constantly displacing each other and reaching a new balanced state. The gas and melt changed rapidly and steadily in the process without sudden changes, especially when the inlet gas pressure was less than 3000 Pa, the change of the viscosity was less than 100 Pa·s, so the melt still did not easily adhere to the wall of the die. At this time, the inlet gas pressure was kept at 1000 Pa, and a stable double gas layer was formed. When we adjusted the inlet gas pressure to continuously decrease, the melt completely extruded the gas from the die and the gas flow channels inside the die were easy to block, so we determined the best inlet gas pressure to be 1000 Pa.

## Figures and Tables

**Table 1:** Physical parameters of the polypropylene (PP, K9015).

Physical Parameters	Melt Point (°C)	Melt Index (g/10 min)	Density (kg/m^3^)	Rockwell Hardness (R)	Tensile Strength (Mpa)
PP, K9015	163	1.6	723	40	14

**Table 2:** Processing parameters.

Die Temperature (°C)	Melt Temperature (°C)	Gas Temperature (°C)	Extruder Speed (r/min)	Pulling System Speed (r/min)
190	190	190	5.5	3

**Table 3:** Numerical simulation parameters of the melt and air.

Param-Eters	τ∗ (Pa)	$D_{1}$ (Pa·s)	$D_{2}$ (K)	$D_{3}$ (K/Pa)	$A_{1}$	$A_{2}$ (K)	n	Thermal Conductivity (W/m·K)	Specific Heat (J/kg·K)
Melt	51948.3	6360	263.15	0	31	51.6	0.2632	0.22	1883
Gas	0	0	0	0	0	0	0	0.037	1026

**Table 4:** Parameters for the Tait equation.

*b*_1_	*b*_2_	*b*_3_	*b*_4_	*b*_5_	*b*_6_
1.316 × 10^−3^	9.947 × 10^−7^	9.51265 × 10^7^	4.804 × 10^−3^	436	7.3 × 10^−8^

## References

[1] Tseng H.H. (1991). Software Design & Experimental Verification of Polymer Flow Through A Pipe Extrusion Die. Ph.D. Thesis.

[2] Zou W.D. (2006). Study on Extrusion Processing of Profile Pipe with Small Cross-Section. Master’s Thesis.

[3] Huang W., Guo Y.C., Wu D.M. (2008). Experimental Study on The Extrusion Processing of PA12 Double-Lumen Catheter. Eng. Plast. Appl..

[4] Li K. (2008). Numerical Analysis and Experimental Study of Polymer Micro Extrusion Flow. Master’s Thesis.

[5] Gong X. (2009). Flow Simulation of Micro Tubing Extrusion Die and Study of Micro Tubing Processing. Master’s Thesis.

[6] Zhao D.Y., Wang M.J., Li K., Song M.C., Jin Y.F. (2010). Flow Uniformity of Polymer Micro Extrusion Processing. Polym. Mater. Sci. Eng..

[7] Zou J., Ling Z.H., Wang Z.Y., Jin Y.F., Zhao D.Y. (2010). Numerical Simulation and Analysis of the Effects of Compression Angle on Flow Uniformity of Double-chamber Micro-Tube. Die Mould Manuf..

[8] Chen Y.W. (2011). Research on the Processing of PA11 Micro-tube Precision Extrusion. Master’s Thesis.

[9] Le G.B., Wang M.J., Zhao D.Y., Tian H.Q. (2012). Experimental Investigation of Extrusion Process of Double-lumen Micro Tube. J. Mech. Eng..

[10] Zheng J.L. (2012). Computer Simulation of Micro-Tube Extrusion Process. Master’s Thesis.

[11] Zheng J.L., Ge D.W., Chen Y.W., Wu H.W. (2012). Variation of Tube Diameter and Wall Thickness in Medical Micro-diameter Tube Extrusion. Plastics.

[12] Xiao X.H. (2013). Analysis and Experimental Study of Polymer Micro Tubing Extrusion Flow. Master’s Thesis.

[13] Chen Y.H. (2013). Analysis of Draw Flow Field and Deformation Control during Extrusion of Polymer Catheter. Master’s Thesis.

[14] Fu Z.H., Yin Y.G., Wang Z.W., Yao C. (2015). Influences of Gas Injection Pressure on Plastic Microtube Extrusion Molding. Chin. Plast. Ind..

[15] Tang D., Fang W.L., Fan X.H., Li D.Y., Peng Y.H. (2014). Effect of Die Design in Microchannel Tube Extrusion. Procedia Eng..

[16] Jin G.B., Wang M.J., Zhao D.Y., Tian H.Q., Jin Y.F. (2014). Design and Experiments of Extrusion Die for Polypropylene Five-Lumen Micro Tube. J. Mater. Process. Technol..

[17] Wang B.X., Wang W., Guo L.H. (2005). Design of An Extrusion Head Used to Produce Tenuous Interventional Catheter Mod. Plast. Process. Appl..

[18] Zhu C.W., Wu D.M. (2009). Inverse Extrudate Swell for Multi-Lumen Precise Medical Catheter. Plastics.

[19] Xu B. (2010). Research on Microscale Effects of Filling Flow and Key Technology of Micro Mold in Micro Injection Molding. Ph.D. Thesis.

[20] Le G.B., Wang M.J., Zhao D.Y., Tian H.Q. (2012). A Design Method of Non-Symmetry Flowing Balance for Right Angle Extrusion Die of Polymeric Double-Lumen Micro-Tube. J. Chem. Ind. Eng..

[21] Xie Y.J. (2013). Design of Extrusion Mould for Polymer Five-lumen Micro Tube Based on Flowing Balance. Master’s Thesis.

[22] Le G.B. (2014). Research on Design and Manufacturing Technology and Extrusion Process of Polymer Micro-extrusion Dies for Interventional Medical Catheters. Ph.D. Thesis.

[23] Jin G.B., Zhao D.Y., Wang M.J. (2015). Study on design and experiments of extrusion die for polypropylene single-lumen micro tubes. Microsyst. Technol..

[24] Housiadas K., Georgiou G., Tsamopoulos J. (2000). The Steady Annular Extrusion of a Newtonian Liquid Under Gravity and Surface Tension. Int. J. Numer. Methods Fluids.

[25] Mitsoulis E. (2007). Annular Extrudate Swell of Newtonian Fluids: Effects of Compressibility and Slip at The Wall. J. Fluids Eng..

[26] Mitsoulis E. (2009). Annular Extrudate Swell of Newtonian Fluids Revisited: Extended Range of Compressible Simulations. J. Fluids Eng..

[27] Karapetsas G., Tsamopoulos J. (2008). Steady Extrusion of Viscoelastic Materials from An Annular Die. J. Non-Newtonian Fluid Mech..

[28] Intawong N., Wiratket A., Meechue P. (2014). Flow Visualization & Extrudate Swell Behavior of Natural Rubber Compound in Annular Die Capillary Rheometer. Polímeros.

[29] Ganvir V., Lele A., Thaokar R. (2009). Prediction of Extrudate Swell in Polymer Melt Extrusion Using an Arbitrary Lagrangian Eulerian (ALE) Based Finite Element Method. J. Non-Newtonian Fluid Mech..

[30] Ganvir V., Gautham B.P., Pol H. (2011). Extrudate Swell of Linear and Branched Polyethylenes: ALE Simulations and Comparison with Experiments. J. Non-Newtonian Fluid Mech..

[31] Konaganti V.K., Ansari M., Mitsoulis E. (2015). Extrudate Swell of a High-Density Polyethylene Melt: II. Modeling Using Integral and Differential Constitutive Equations. J. Non-Newtonian Fluid Mech..

[32] Delgadillo-Velázquez O., Georgiou G., Sentmanat M. (2008). Sharkskin and Oscillating Melt Fracture: Why in Slit and Capillary Dies and Not in Annular Dies?. Polym. Eng. Sci..

[33] Mitsoulis E., Georgiou G.C., Kountouriotis Z. (2012). A Study of Various Factors Affecting Newtonian Extrudate Swell. Comput. Fluids.

[34] Burghelea T.I., Griess H.J., Münstedt H. (2010). Comparative Investigations of Surface Instabilities (“Sharkskin”) Of A Linear and A Long-Chain Branched Polyethylene. J. Non-Newtonian Fluid Mech..

[35] Ganß M., Satapathy B.K., Thunga M. (2008). Structural Interpretations of Deformation and Fracture Behavior of Polypropylene/Multi-Walled Carbon Nanotube Composites. Acta Mater..

[36] Brzoskowski R., White J.L., Szydlowski W. (1987). Air-Lubricated Die for Extrusion of Rubber Compounds. Rubber Chem. Technol..

[37] Poslinski A.J., Oehler P.R., Stokes V.K. (1995). Isothermal Gas-Assisted Displacement of Viscoplastic Liquids In Tubes. Polym. Eng. Sci..

[38] Liang R.F., Mackley M.R. (2001). The Gas-Assisted Extrusion of Molten Polyethylene. J. Rheol..

[39] Xu Y.Q., Huang X.Y., Liu H.S. (2015). Numerical Simulation of Die Swell in Gas-assisted Pipe Extrusion. Eng. Plast. Appl..

[40] Hatzikiriakos S.G., Dealy J.M. (1992). Wall Slip of Molten High Density Polyethylenes. II. Capillary Rheometer Studies. J. Rheol..

[41] Stewart C.W. (1993). Wall Slip in The Extrusion of Linear Polyolefins. J. Rheol..

[42] Adjari A., Brochard-Wyart F., de Gennes P.G. (1994). Slippage of An Entangled Polymer Melt on A Grafted Surface. Physica A.

[43] Wang S.Q., Drda P.A. (1996). Superfluid-Like Stick− Slip Transition in Capillary Flow of Linear Polyethylene Melts. 1. General Features. Macromolecules.

[44] Durand V., Vergnes B., Agassant J.F. (1996). Experimental Study and Modeling of Oscillating Flow of High Density Polyethylenes. J. Rheol..

[45] Robert L., Demay Y., Vergnes B. (2004). Stick-Slip Flow of High Density Polyethylene In A Transparent Slit Die Investigated By Laser Doppler Velocimetry. Rheol. Acta.

[46] Park H.E., Lim S.T., Smillo F. (2008). Wall Slip and Spurt Flow of Polybutadiene. J. Rheol..

[47] Hatzikiriakos S.G. (2012). Wall Slip of Molten Polymers. Prog. Polym. Sci..

[48] Damianou Y., Philippou M., Kaoullas G. (2014). Cessation of Viscoplastic Poiseuille Flow with Wall Slip. J. Non-Newtonian Fluid Mech..

[49] Ren Z., Huang X.Y., Liu H.S., Deng X.Z. (2016). Numerical Simulation of Polymer Gas-Assisted Extrusion Based on Gas/Liquid Two-Phase Flow. Polym. Mater. Sci. Eng..

[50] Ren Z., Huang X.Y., Liu H.S., Deng X.Z. (2016). Experiment and Simulation on the Effect of Gas Pressure on Polymer Gas Assisted Extrusion Forming. J. Sichuan Univ..

[51] Ren Z., Huang X.Y., Liu H.S. (2016). Three Dimensional Viscoelastic Numerical Simulation Of Gas-Assisted Extrusions For Hollow Profile Polymer Melt. J. Cent. South Unive. Nat. Sci..

[52] Ren Z. (2017). Theoretical and Experimental Study on Gas-assisted Extrusion Forming of Plastic Micro-tubes. Ph.D. Thesis.

[53] Ren Z., Huang X.Y., Liu H.S. (2017). Wall Slip Velocity Measurement of Molten Polypropylene in Capillary Flow Based on Length-Corrected Mooney Technique. J. Appl. Polym. Sci..

[54] Ren Z., Huang X.Y., Liu H.S. (2016). A Novel Unified Wall Slip Model for P Oiseuille Flow of Polymer Melt in The Circular Tube. Polym. Eng. Sci..

[55] Gu J.H., Li Y.L., Song L., Xie C.P. (2014). Principle and Device for Polymer PVT Property Testing. Plastics.

[56] Li F., Sun J., Li Y.Q. (2012). Analysis of Rheological Properties and PVT Relationships of Polypropylene in Injection molding. China Plast..

[57] Huang X.Y., Liu H.S., Zhou G.F. (2005). Experiment and Simulation for Extrusion Swell in Gas-assisted Extrusion. Plastics.

[58] Huang X.Y. (2006). Theoretical and Experimental Study of Polymer Gas-assisted Extrusion from Dies. Ph.D. Thesis.

[59] Huang Y.B. (2011). Theoretical and Experimental Study on Polymer Gas-assisted Co-extrusion. Ph.D. Thesis.

[60] Deng X.Z. (2014). Experimental and Theoretical Study on Gas-assisted Co-extrusion of Plastic Profile with an Irregular Cross-section. Ph.D. Thesis.

